# Can a Higher Protein/Low Glycemic Index vs. a Conventional Diet Attenuate Changes in Appetite and Gut Hormones Following Weight Loss? A 3-Year PREVIEW Sub-study

**DOI:** 10.3389/fnut.2021.640538

**Published:** 2021-03-22

**Authors:** Marion E.C. Buso, Radhika V. Seimon, Sally McClintock, Roslyn Muirhead, Fiona S. Atkinson, Shannon Brodie, Jarron Dodds, Jessica Zibellini, Arpita Das, Anthony L. Wild-Taylor, Jessica Burk, Mikael Fogelholm, Anne Raben, Jennie C. Brand-Miller, Amanda Sainsbury

**Affiliations:** ^1^Division of Human Nutrition and Health, Wageningen University, Wageningen, Netherlands; ^2^The Boden Collaboration for Obesity, Nutrition, Exercise, and Eating Disorders, Faculty of Medicine and Health, Charles Perkins Centre, The University of Sydney, Camperdown, NSW, Australia; ^3^School of Life and Environmental Sciences and Charles Perkins Centre, The University of Sydney, Camperdown, NSW, Australia; ^4^Department of Food and Environmental Sciences, University of Helsinki, Helsinki, Finland; ^5^Department of Nutrition, Exercise and Sports, University of Copenhagen, Copenhagen, Denmark; ^6^Steno Diabetes Center Copenhagen, Copenhagen, Denmark; ^7^School of Human Sciences, Faculty of Science, The University of Western Australia, Crawley, WA, Australia

**Keywords:** weight maintenance, appetite, gut hormones, ghrelin, peptide YY

## Abstract

**Background:** Previous research showed that weight-reducing diets increase appetite sensations and/or circulating ghrelin concentrations for up to 36 months, with transient or enduring perturbations in circulating concentrations of the satiety hormone peptide YY.

**Objective:** This study assessed whether a diet that is higher in protein and low in glycemic index (GI) may attenuate these changes.

**Methods:** 136 adults with pre-diabetes and a body mass index of ≥25 kg/m^2^ underwent a 2-month weight-reducing total meal replacement diet. Participants who lost ≥8% body weight were randomized to one of two 34-month weight-maintenance diets: a higher-protein and moderate-carbohydrate (CHO) diet with low GI, or a moderate-protein and higher-CHO diet with moderate GI. Both arms involved recommendations to increase physical activity. Fasting plasma concentrations of total ghrelin and total peptide YY, and appetite sensations, were measured at 0 months (pre-weight loss), at 2 months (immediately post-weight loss), and at 6, 12, 24, and 36 months.

**Results:** There was a decrease in plasma peptide YY concentrations and an increase in ghrelin after the 2-month weight-reducing diet, and these values approached pre-weight-loss values by 6 and 24 months, respectively (*P* = 0.32 and *P* = 0.08, respectively, vs. 0 months). However, there were no differences between the two weight-maintenance diets. Subjective appetite sensations were not affected by the weight-reducing diet nor the weight-maintenance diets. While participants regained an average of ~50% of the weight they had lost by 36 months, the changes in ghrelin and peptide YY during the weight-reducing phase did not correlate with weight regain.

**Conclusion:** A higher-protein, low-GI diet for weight maintenance does not attenuate changes in ghrelin or peptide YY compared with a moderate-protein, moderate-GI diet.

**Clinical Trial Registry:** ClinicalTrials.gov registry ID NCT01777893 (PREVIEW) and ID NCT02030249 (Sub-study).

## Introduction

Overweight and obesity are rapidly increasing worldwide, with the number of people with obesity having more than doubled since 1980 (1). In 2016, 671 million adults worldwide were classified as having obesity (2). Overweight and obesity have multiple adverse health consequences, such as cardiovascular diseases and type 2 diabetes, which are the leading causes of death related to high body mass index (3). To mitigate these diseases, lifestyle management of obesity is necessary. For people with obesity and diabetes (or a high risk of diabetes), even moderate weight reduction of 5–10% of initial weight improves glycemic control (or reduces the risk of developing diabetes) (4, 5). However, lifestyle-based weight-loss interventions are often ineffective in the long term, resulting in regain of all of the weight lost in about 50% of individuals by 5 years (6).

There are many possible mechanisms for the difficulty in maintaining a reduced weight. These include physiological adaptations, such as decreased energy expenditure and increased hunger sensations (7–10), associated with increases in circulating concentrations of the appetite-stimulating hormone, ghrelin, and reductions in that of the appetite-suppressing hormone, peptide YY (11–16). These changes in appetite sensations and gut hormone concentrations after weight loss can persist for 1 year (17–19) to 3 years (20) after weight loss in people who originally had a body mass index in the overweight or obese range, providing a possible explanation for the low success rate of weight maintenance at a reduced body weight. Finding ways to reduce appetite after weight loss could potentially help to increase the long-term success of weight-loss interventions.

Some evidence shows that reducing appetite sensations might be achievable with a weight-loss or weight-maintenance diet that is higher in protein or lower in glycemic index (GI) than the diet that is conventionally recommended for health (21–24). For instance, a 20% higher protein intake (18 vs. 15% of energy) during a weight-maintenance diet after weight loss caused a 50% lower weight regain 3 months after weight loss, in association with higher sensations of satiety (23). Comparatively, a diet with a low GI seems to increase satiety in the majority of short-term studies (21, 25). Moreover, a low-GI diet was more effective for maintenance of a reduced body weight, when combined with a higher protein intake and when compared to a diet of moderate-GI and/or moderate-protein content, as demonstrated in the 26-week Diogenes study (26).

In light of the above considerations, it is possible that a diet that is higher in protein and low in GI may be able to attenuate the increase in appetite and associated changes in circulating gut hormone concentrations seen with weight loss and may therefore help to promote weight-loss maintenance in the long term. To our knowledge, this has never been tested in a randomized control trial. Therefore, the aim of this study was to evaluate if the appetite-inducing effect of weight loss can be reversed by a higher-protein, low-GI vs. a moderate-protein, medium-GI weight-maintenance diet. This was tested in a sub-study of the PREVIEW (PREVention of diabetes through lifestyle Intervention and population studies in Europe and around the World) Study in Sydney, Australia.

## Materials and Methods

### Participants and Setting

Participants in this sub-study were investigated at the Charles Perkins Centre Royal Prince Alfred Clinic on the University of Sydney campus in Camperdown, New South Wales, Australia. Adults with pre-diabetes [defined by (i) plasma glucose concentrations of 5.6–6.9 mmol/L when fasted and/or (ii) impaired glucose tolerance, with plasma glucose concentrations of 7.8–11.0 mmol/L at 2 h after oral administration of a standard 75-g glucose dose and a fasting plasma glucose concentration of <7.0 mmol/L] and a body mass index of ≥25 kg/m^2^ were eligible for the PREVIEW randomized controlled trial in Sydney. Of those participants that started the trial, those who lost 8% or more of their initial body weight during the 2-month weight-reducing diet were eligible to start the subsequent weight-maintenance diet. Full details of the selection criteria for the PREVIEW randomized controlled trial have been published previously (27). All participants gave written informed consent prior to participating in the PREVIEW randomized controlled trial and the current sub-study.

### Design

The PREVIEW Study was a multinational 3-year study whose goal was to identify the most efficient lifestyle factors for the prevention of diabetes in a population of people with pre-diabetes. The randomized controlled trial of the PREVIEW Study involved a 36-month intervention, commencing with a weight-reducing phase (2 months) followed by a weight-maintenance phase (34 months) as detailed below.

### Weight-Reducing and Weight-Maintenance Phases

The 2-month weight-reducing phase entailed a total meal replacement diet delivering ~3,400 kJ (800 kcal) per day, comprising 15–20% of energy from fat (E% fat), 35–40 E% protein, and 45–50 E% carbohydrate (CHO) (Cambridge Weight Plan^©^, Ltd, UK). During the weight-maintenance phase, participants were randomly assigned to either a higher-protein (25 E%) and moderate-CHO (45 E%) diet with a low-GI (GI≤50) (HP/LGI) or moderate-protein (15 E%) and higher-CHO (55 E%) diet with a moderate GI (GI≥56) (MP/MGI). Both diets were moderate in fat (30 E%). Participants were not given specific targets for energy intake but were instead encouraged to “eat to appetite” and to follow a regular meal pattern, with no other specifications about the timing of eating. In other words, the weight-maintenance diets were *ad libitum* for energy. In addition to randomization for weight-maintenance diets, participants were randomized to one of two physical activity interventions: either a high-intensity exercise intervention, or a moderate-intensity exercise intervention. Due to the small sample size of this sub-study (136 participants), the impact of physical activity interventions will not be presented in this paper, albeit analyses of effects of the two weight-maintenance diets will be adjusted for the exercise intervention group.

### Outcomes and Time Points

For the sub-study reported in this paper, the following outcomes were measured and analyzed at the following time points: 0 months (pre-weight loss), 2 months (immediately post-weight loss, which is at the start of the weight-maintenance phase), and 6, 12, 24, and 36 months (end of the weight-maintenance phase). Participants were asked to attend our clinical research facility in the morning, having consumed nothing but water since midnight. They then underwent different measurements, as described in detail elsewhere (27). Measurements relevant or specific to this sub-study are detailed below.

### Anthropometric Measurements

Body weight was measured to the nearest 0.1 kg using a calibrated Tanita BWB-800 digital scale (Wedderburn) and with participants in light clothing without shoes. Height was measured to the nearest 0.5 cm with a Harpenden 602VR Stadiometer (Holtain Limited).

### Fasting Appetite Sensations

Fasting sensations of hunger, desire to eat, prospective consumption (how much food participants felt they could eat at that time), and fullness were quantified using visual analog scales printed on paper (28). Each visual analog scale consisted of a 100-mm horizontal line, where 0 represented “sensation not felt at all” and 100 represented “sensation felt the greatest.” Participants were asked to place a vertical mark along each horizontal line to indicate the strength of the sensation they felt at that particular time. The intensity of the sensation (distance from 0) was then measured with a ruler, generating a score in the range of 0–100 mm.

### Blood Collection and Sample Storage

For measurement of fasting plasma concentrations of ghrelin and peptide YY, blood samples were collected into ice-cold EDTA-treated tubes (Becton Dickinson). Immediately after collection, samples were plunged up to the neck into wet ice. Plasma was then obtained by centrifugation of blood samples at 3,200 rpm (~1,600 × g) for 10 min at 4°C (Eppendorf Centrifuge 5702, Eppendorf AG). Plasma aliquots were pipetted into CryoPure® tubes (Sarstedt Australia) and immediately stored at −80°C until analyzed.

### Fasting Plasma Concentrations of Ghrelin and Peptide YY

Concentrations of ghrelin (total) and peptide YY (total) in plasma samples were measured using commercial radioimmunoassay kits (Merck Millipore, Billerica, MA, USA) according to the manufacturer's instructions. These assays detect minimum concentrations of 93 pg/ml ghrelin and 20 pg/ml peptide YY. Inter- and intra-assay coefficients of variability (%CV) were, respectively, 4.0 and 5.4% for ghrelin and 15.0 and 4.6% for peptide YY.

### Statistical Analyses

All analyses were by intention-to-treat and were performed in Statistical Analysis System (SAS) version 9.4 (SAS Institute, North Carolina, US). Participant characteristics at 0 months (pre-weight loss) were evaluated both in participants who came to the final 36-month outcome measurement (“completers”) and in those who did not (“non-completers”) and are presented as means (with standard deviation) or—when data were skewed—as medians [with interquartile range]. To compare completers and non-completers, Student *t*-tests were used for normally distributed variables, Mann–Whitney–Wilcoxon non-parametric tests were used for skewed variables, and Chi-square tests were used for categorical variables. To investigate between-group effects of the weight-maintenance diets over time, intention-to-treat analyses were performed on all raw outcomes using a repeated constrained linear mixed model from 2 months (immediately post-weight loss, which is at the start of the weight-maintenance phase), with a restricted maximum likelihood. With this model, the mean of the 2-month (post-weight loss/baseline) values is constrained (assumed) to be the same for all groups due to the process of randomization (29). Modeling of the covariance structure was undertaken to account for the dependent nature of the data at different time points. An unstructured or spatial power (SP[POW]) covariate structure was used (because time points were not equally spaced), and models with the best fit were presented. Plasma ghrelin and peptide YY concentrations were log-transformed to improve the fit of the models (assumption of normality and variance homogeneity of the residuals were met). The repeated constrained linear mixed models included the following variables: age, sex, pre-weight loss (0-month) value, the exercise group to which each participant had been assigned, and the time^*^diet interaction. As a secondary analysis, and because of lack of differences between the two weight-maintenance diets, repeated linear mixed models were fitted to evaluate changes within the entire population and to compare the data from each time point (i.e., 2, 6, 24, and 36 months) with the value at 0 months (pre-weight loss, before the weight-reducing diet). Bonferroni corrections were used to account for the multiple comparisons.

To investigate whether changes in plasma gut hormone concentrations correlated with changes in weight during the weight-reducing diet (as measured from 0 to 2 months) and during the weight-maintenance diets (as measured between 2 and 36 months), we undertook correlation analyses with Spearman's rank correlation. *P*-values <0.05 (or <0.01 when Bonferroni adjustments were applied) were considered statistically significant.

## Results

**Figure 1:**
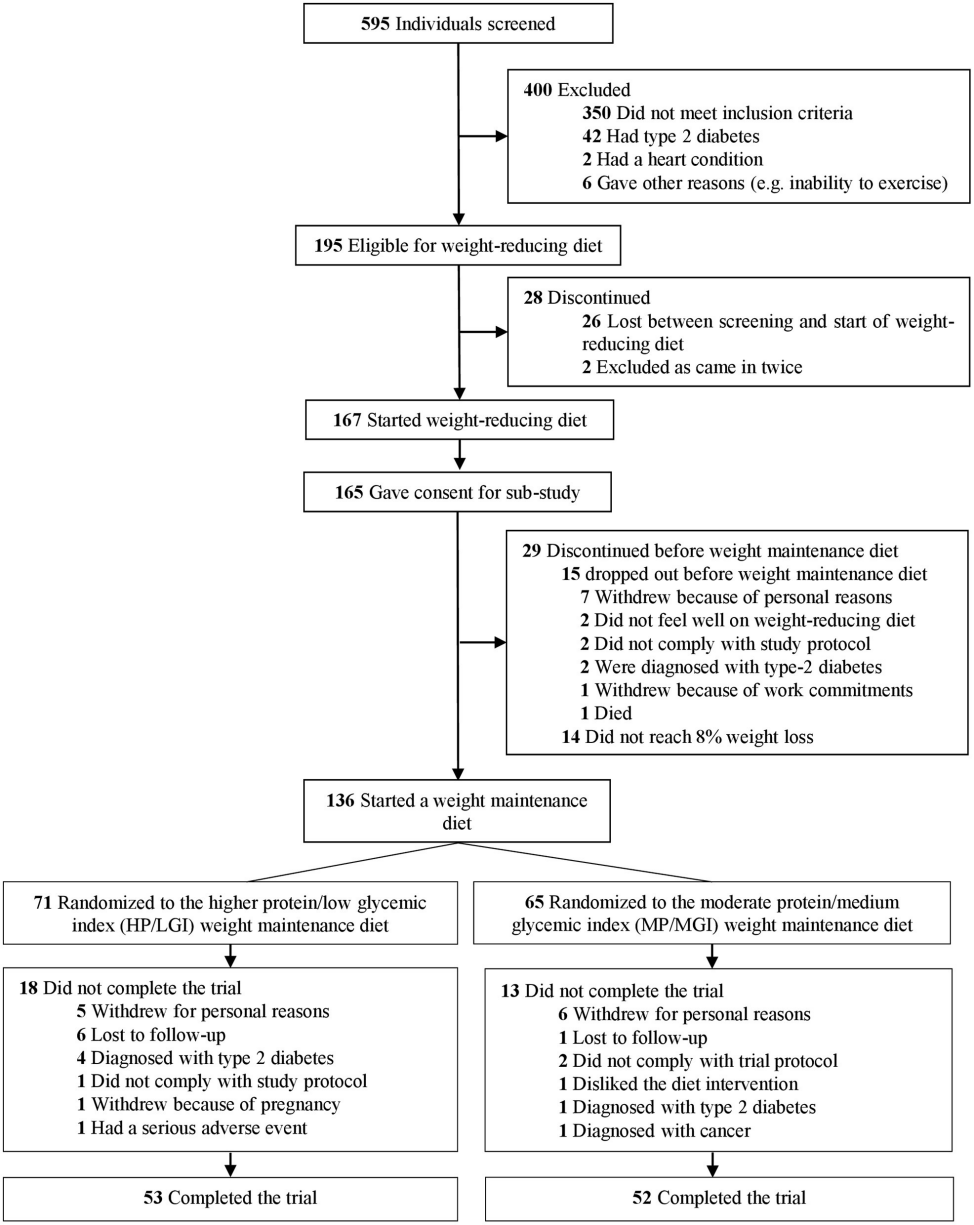
Trial flow diagram. The trial involved a 2-month weight-reducing diet consisting of a total meal replacement diet with a prescribed daily energy intake of 3,400 kJ (800 kcal). As per the registered trial design, participants who lost 8% or more of their initial body weight proceeded to a 34-month weight-maintenance diet involving either a diet with a higher protein (25% of energy) and moderate carbohydrate content (45% of energy) with a low glycemic index (≤50) (HP/LGI) vs. a diet with a moderate protein (15% of energy) and higher carbohydrate content (55% of energy) with a moderate GI (≥56) (MP/MGI).

In total, 136 participants achieved 8% or greater weight loss at the end of the weight-reducing phase and were eligible to be randomized to one of the two weight-maintenance diets (Figure 1). Characteristics of participants pre-weight loss (i.e., at 0 months) are presented in Table 1. Overall, 68.4% of the participants were female (*n* = 93/136). Participants had a median [interquartile range, IQR] age of 56 [48–62] years, a median [IQR] body weight of 100.2 [87.2–112.1] kg, and a median [IQR] body mass index of 34.8 [31.4–39.2] kg/m^2^. In total, 105 of the 136 participants at the Sydney site (77.2%) completed the trial to the final (36-month) time point, 53 out of 71 (74.6%) from the HP/LGI weight-maintenance diet group, and 52 out of 65 (80.0%) from the MP/MGI weight-maintenance diet group, as detailed in Figure 1. There were differences in body weight and body mass index between completers and non-completers before weight loss, with completers having a lower body weight (99.4 vs. 108.4 kg, *P* = 0.04) and body mass index (33.9 vs. 37.7 kg/m^2^, *P* = 0.02) compared to non-completers at 0 months. There were no statistically significant differences between completers and non-completers within each study arm (all *P*-values >0.05); however, median pre-weight-loss values were lower for ghrelin (861 vs. 930 pg/ml, *P* = 0.33), but higher for hunger (31.0 vs. 20.5 mm, *P* = 0.10) and desire to eat (37 vs. 25 mm, *P* = 0.13) in the completers group vs. the non-completers group within the HP/LGI group only (Table 1).

**Table 1:** Characteristics before weight loss of all participants that completed the 2-month weight-reducing diet and lost ≥8% of their initial body weight, subdivided into those who completed or did not complete the subsequent 34-month weight-maintenance diet to which they were randomized [either a higher protein/low GI (HP/LGI) diet or a moderate protein/moderate GI (MP/MGI) diet].

	**Both weight-maintenance diets**			**HP/LGI**			**MP/MGI**		
**Characteristic^a^**	**All** **participants** **(*n* = 136)**	**Completers** **(*n* = 105)**	**Non-completers** **(*n* =31)**	**All participants** **(*n* = 71)**	**Completers^d^** **(*n* = 53)**	**Non-completers^d^** **(*n* = 18)**	**All participants** **(*n* = 65)**	**Completers^e^** **(*n* = 52)**	**Non-completers^e^** **(*n* = 13)**
**General**									
Female, % (n)	68.4 (93)	66.7 (70)	74.2 (23)	69.0 (49)	67.9 (36)	72.2 (13)	67.7 (44)	65.4 (34)	76.9 (10)
Age, years	56 [48–62]	56 [50–62]	53 [45–59]	53 [45–62]	57 [45–62]	53 [39–60]	55 [50–61]	56 [50–62]	55 [48–58]
**Anthropometrics**									
Height, m	1.68 (0.09)	1.68 (0.09)	1.67 (0.08)	1.67 (0.09)	1.68 (0.09)	1.67 (0.08)	1.68 (0.09)	1.68 (0.09)	1.67 (0.08)
Weight, kg	100.2 [87.2–112.1]	99.4 [85.9–105.6]	108.4 [93.2–116.5]	100.1 [89.4–113.3]	99.4 [85.4–104.4]	104.6 [93.2–114.8]	100.8 [86.3–111.1]	99.0 [86.1–106.9]	113.1 [93.7–118.3]
Body mass index, kg/m^2^	34.8 [31.4–39.2]	33.9 [31.1–37.8]	37.7 [31.9–43.4]	35.0 [31.9–38.7]	34.9 [31.6–37.6]	36.9 [32.3–43.4]	33.9 [31.2–39.7]	33.5 [30.8–39.0]	40.4 [31.8–44.9]
Weight loss at 2 months, %	11.0 [9.8–13.0]	11.1 [9.9–13.0]	10.5 [9.7–12.9]	11.1 [9.8–12.9]	11.2 [10.2–12.7]	10.3 [9.7–12.9]	10.9 [9.7–13.0]	11.0 [9.8–13.0]	10.8 [9.1–12.7]
Weight loss at 2 months, kg	11.0 [9.1–13.3]	10.8 [9.1–13.2]	11.3 [9.4–14.4]	11.2 [9.4–13.5]	11.3 [9.4–13.5]	11.2 [9.4–12.7]	10.7 [9.1–12.8]	10.7 [9.0–12.8]	12.3 [9.7–14.4]
**Fasting plasma gut hormone concentrations and appetite sensations**									
Ghrelin, pg/ml^b^	859 [679–1240]	848 [625–1249]	891 [737–1224]	880 [649–1224]	861 [577–1164]	930 [767–1224]	824 [685–1307]	823 [708–1396]	825 [546–1307]
Peptide YY, pg/ml^b^	249 [197–305]	236 [191–306]	257 [209–300]	255 [203–318]	253 [197–322]	260 [209–296]	233 [181–293]	232 [181–290]	255 [187–384]
Hunger, mm^c^	30.0 [14.0–49.0]	30.5 [16.0–50.0]	27.0 [11.0–45.0]	30.0 [14.0–49.0]	31.0 [15.0–49.0]	20.5 [9.0–44.0]	30.0 [20.0–51.5]	30.0 [19.0–52.0]	30.0 [21.0–45.0]
Desire to eat, mm^c^	36.5 [15.5–58.0]	41.0 [16.0–60.0]	25.0 [14.0–46.0]	32.0 [15.0–60.0]	37.0 [15.0–61.0]	25.0 [13.0–44.0]	41.0 [16.0–57.0]	44.0 [15.0–57.5]	34.0 [23.0–47.0]
Prospective consumption, mm^c^	43.5 [27.5–54.5]	45.0 [25.0–54.0]	39.0 [18.0–63.0]	41.0 [25.0–53.0]	42.0 [27.0–53.0]	40.0 [18.0–49.0]	46.0 [30.0–55.0]	46.5 [31.0–54.5]	37.0 [23.0–69.0]
Fullness, mm^c^	32.5 [16.0–51.5]	33.0 [16.0–50.0]	30.0 [9.0–52.0]	29.0 [52.0–36.0]	29.0 [16.0–53.0]	32.0 [14.0–51.0]	33.8 [12.0–49.0]	33.5 [12.0–49.0]	30.0 [6.0–57.0]

a *Mean (standard deviation), median [25–75th percentile] or % (n)*.

b *Valid data on plasma hormone concentrations are available for 93 participants*.

c *Data for one participant is missing because the participant did not complete the questionnaire*.

d *Valid data on plasma hormone concentrations are available data for 33 participants in completers and 14 participants in non-completers in the HP/LGI diet*.

e *Valid data on plasma hormone concentrations are available data for 35 participants in completers and 11 participants in non-completers in the MP/MGI diet*.

### Body Weight

**Figure 2:**
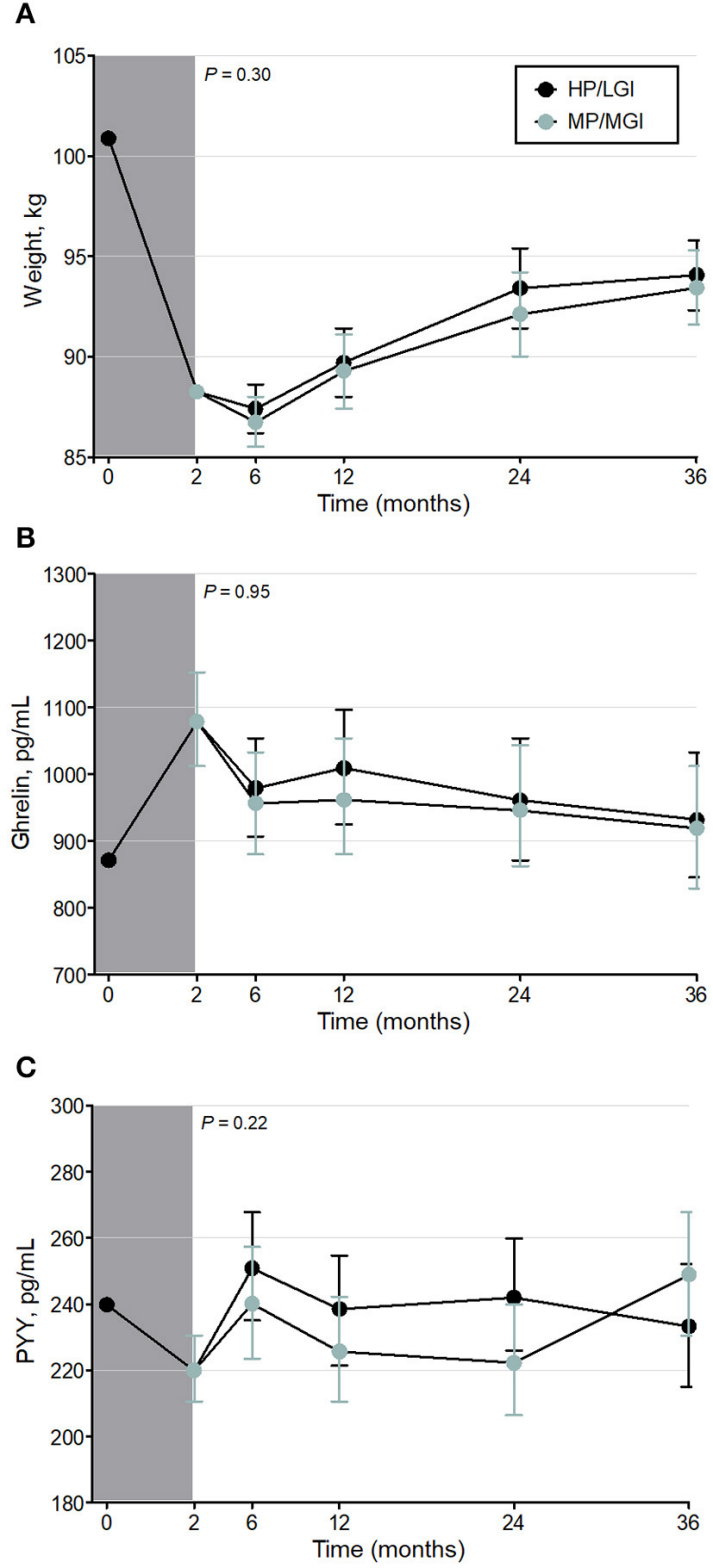
Weight **(A)** and fasting plasma gut hormone concentrations [Ghrelin **(B)** and Peptide YY **(C)**] before, during, and after the weight-reducing (gray shaded bar) and weight-maintenance diets of the PREVIEW sub-study [either a higher protein/low GI (HP/LGI) or a moderate-protein/moderate-GI (MP/MGI) weight-maintenance diet]. Results of a constrained linear mixed model (2–36 months) are shown as estimated marginal means (i.e., means adjusted for age, sex, exercise group, and the value at 0 months) ± 95% CI. There were no statistically significant differences between the weight-maintenance diets at any time point (*P-*values for the interaction of diet*time are shown on each panel). Ghrelin and peptide YY were log-transformed in the analyses.

At the end of the weight-reducing phase, median [IQR] weight loss in all participants was 11.0 [9.8–13.0]% of initial body weight (Table 1). Weight loss was similar between completers and non-completers, with a median [IQR] weight loss of 11.1 [9.9–13.0]% vs. 10.5 [9.7–12.9]% of initial body weight, respectively, *P* = 0.32. During the weight-maintenance diets, there were no observed differences in weight between groups (*P* = 0.30) (Figure 2A and Table 2). At 36 months, the mean difference (95% CI) between groups was 0.62 (−1.87–3.12) kg. Overall, participants experienced partial weight regain, with body weight remaining lower than pre-weight-loss (0-month) values until the end of the intervention (*P* < 0.001 at all time points, Table 3). On average, participants had a mean overall regain of 48.7% of the weight lost.

**Table 2:** Body weight, plasma gut hormone concentrations, and fasting appetite sensations with the higher-protein/low-GI (HP/LGI) and moderate-protein/moderate-GI (MP/MGI) weight-maintenance diets.

**Measurement**	**No**.	**HP/LGI** **estimated marginal means (95% CI)**	**No**.	**MP/MGI** **estimated marginal means (95% CI)**	**Mean between-group difference (95% CI)**	***P*-value^a^**
**Weight, kg**						0.30
2 mo (all)	136	88.2 (87.9–88.6)		–	–	
6 mo	67	87.4 (86.2–88.6)	59	86.7 (85.5–88.0)	0.66 (−0.93–2.27)	
12 mo	64	89.7 (88.0–91.4)	55	89.3 (87.4–91.1)	0.41 (−2.06–2.89)	
24 mo	61	93.4 (91.4–95.4)	53	92.1 (90.0–94.2)	1.29 (−1.60–4.18)	
36 mo	53	94.1 (92.3–95.8)	52	93.4 (91.6–95.3)	0.62 (−1.87–3.12)	
**Ghrelin, log pg/ml**						0.95
2 mo (all)	93	6.98 (6.92–7.05)				
6 mo	42	6.89 (6.81–6.96)	37	6.86 (6.78–6.94)	0.02 (−0.07–0.11)	
12 mo	41	6.92 (6.83–7.00)	38	6.87 (6.78–6.96)	0.05 (−0.07–0.17)	
24 mo	39	6.87 (6.77–6.96)	36	6.85 (6.76–6.95)	0.02 (−0.12–0.15)	
36 mo	33	6.84 (6.74–6.94)	35	6.82 (6.72–6.92)	0.01 (−0.13–0.15)	
**Peptide YY, log pg/ml**						0.22
2 mo (all)	93	5.39 (5.35–5.44)				
6 mo	42	5.52 (5.46–5.59)	37	5.48 (5.41–5.55)	0.04 (−0.04–0.13)	
12 mo	41	5.47 (5.40–5.54)	38	5.42 (5.35–5.49)	0.06 (−0.04–0.15)	
24 mo	39	5.49 (5.42–5.56)	36	5.40 (5.33–5.48)	0.09 (−0.02–0.19)	
36 mo	33	5.45 (5.37–5.53)	35	5.52 (5.44–5.59)	−0.06 (−0.18–0.05)	
**Hunger, mm**						0.98
2 mo (all)	135	34.7 (30.4–38.9)		
6 mo	63	33.9 (28.3–39.6)	56	35.0 (29.1–41.0)	−1.1 (−8.9–6.8)	
12 mo	64	38.2 (32.1–44.3)	55	40.2 (33.6–46.8)	−2.0 (−10.7–6.7)	
24 mo	61	39.5 (33.2–45.7)	53	40.4 (33.7–47.1)	−0.9 (−9.9–8.2)	
36 mo	53	37.6 (31.4–43.9)	52	40.1 (33.7–46.5)	−2.5 (−11.2–6.2)	
**Desire to eat, mm**						0.75
2 mo (all)	135	42.9 (38.8–47.0)		
6 mo	64	40.0 (35.0–45.0)	57	42.1 (36.9–47.3)	−4.2 (−13.0–4.6)	
12 mo	64	43.4 (37.7–49.1)	55	44.7 (38.1–51.2)	−0.76 (−8.9–7.3)	
24 mo	61	43.4 (37.7–49.1)	53	44.1 (38.1–50.2)	1.4 (−6.9–9.7)	
36 mo	53	41.3 (35.3–47.3)	52	39.8 (33.7–45.9)	−2.1 (−9.0–4.73)	
**Prospective consumption, mm**						0.99
2 mo (all)	135	41.1 (38.0–44.2)				
6 mo	64	43.4 (39.8–47.1)	57	43.0 (39.2–46.8)	0.4 (−4.3–5.2)	
12 mo	64	43.3 (39.0–47.6)	55	43.3 (38.7–47.9)	−0.0 (−5.9–5.8)	
24 mo	61	45.1 (40.6–49.5)	53	44.7 (39.9–49.4)	0.4 (−5.7–6.5)	
36 mo	53	42.4 (37.5–47.2)	52	40.5 (35.6–45.4)	1.8 (−4.9–8.6)	
**Fullness, mm**						0.26
2 mo (all)	135	33.7 (29.9–37.6)				
6 mo	64	29.7 (24.2–35.2)	57	36.4 (30.6–42.1)	−6.6 (−14.1–0.8)	
12 mo	64	33.8 (27.9–39.8)	55	33.7 (27.4–40.1)	0.1 (−8.5–8.7)	
24 mo	61	33.5 (27.9–39.1)	53	39.0 (33.1–45.0)	−5.5 (−13.5–2.4)	
36 mo	53	38.8 (32.5–45.1)	52	39.3 (32.9–45.7)	−0.5 (−9.2–8.1)	

a P-values for the interaction of diet*time in the constrained linear mixed model (2–36 months), adjusted for age, sex, physical activity, and value at 0 months (pre-weight loss).

**Table 3:** Body weight, plasma gut hormone concentration and fasting appetite sensations of participants in both groups, pooled into a single group.

**Measurement**	**No**.	**Estimated pooled means** **(95% CI)**	**Mean difference from** **pre-weight loss (0-mo) values (95% CI)**	***P*-value^a^**
**Weight, kg**				
0 mo	136	99.6 (96.3–103.0)	–	–
2 mo	136	88.1 (85.1–91.2)	−11.5 (−12.0 to −11.0)	<0.001
6 mo	126	87.0 (83.9–90.1)	−12.7 (−13.6 to −11.7)	<0.001
12 mo	119	89.4 (86.2–92.6)	−10.3 (−11.6 to −8.9)	<0.001
24 mo	114	92.7 (89.3–96.1)	−6.9 (−8.4 to −5.5)	<0.001
36 mo	105	93.7 (90.4–96.9)	−6.0 (−7.3 to −4.7)	<0.001
**Ghrelin, log pg/ml**				
0 mo	93	6.76 (6.65–6.86)	–	–
2 mo (all)	93	6.99 (6.88–7.09)	0.23 (0.19–0.27)	<0.001
6 mo	79	6.88 (6.77–6.98)	0.12 (0.05–0.19)	0.001
12 mo	79	6.90 (6.79–7.00)	0.14 (0.04–0.23)	0.004
24 mo	75	6.87 (6.75–6.98)	0.11 (−0.01–0.23)	0.08
36 mo	68	6.83 (6.71–6.95)	0.07 (−0.07–0.21)	0.31
**Peptide YY, log pg/ml**				
0 mo	93	5.49 (5.43–5.55)	–	–
2 mo	93	5.41 (5.35–5.47)	−0.08 (−0.12 to −0.04)	<0.0001
6 mo	79	5.52 (5.46–5.59)	0.03 (−0.03–0.09)	0.32
12 mo	79	5.46 (5.4–5.53)	−0.03 (−0.10–0.05)	0.49
24 mo	75	5.45 (5.38–5.52)	−0.04 (−0.13–0.05)	0.39
36 mo	68	5.49 (5.42–5.56)	0.0 (−0.09–0.10)	0.95
**Hunger, mm**				
0 mo	135	34.2 (30–38.4)	–	–
2 mo	135	33.9 (29.1–38.6)	−0.4 (−4.9–4.2)	0.88
6 mo	119	33.8 (29–38.7)	−0.4 (−4.8–4.1)	0.86
12 mo	119	38.4 (33.3–43.5)	4.2 (−0.6–8.9)	0.08
24 mo	114	38.9 (33.8–44)	4.7 (−0.2–9.5)	0.06
36 mo	105	38.3 (33.5–43)	4.0 (−0.9–9)	0.11
**Desire to eat, mm**				
0 mo	136	39.6 (34.9–44.2)	–	–
2 mo	135	42.2 (37.7–46.7)	2.6 (−2.3–7.5)	0.29
6 mo	121	40.2 (35.9–44.5)	0.6 (−3.7–4.9)	0.77
12 mo	119	41.6 (36.6–46.6)	2.0 (−3.1–7.2)	0.43
24 mo	114	43.1 (38.5–47.6)	3.5 (−1.6–8.6)	0.17
36 mo	105	40.0 (35.5–44.6)	0.5 (−4.8–5.7)	0.86
**Prospective consumption, mm**				
0 mo	136	43.4 (39.6–47.2)	–	–
2 mo	135	41.3 (37.7–45)	−2.0 (−5.5–1.4)	0.25
6 mo	121	43.5 (40.1–46.9)	0.2 (−3.2–3.5)	0.93
12 mo	119	43.6 (39.7–47.4)	0.2 (−3.5–3.9)	0.91
24 mo	114	45.2 (41.3–49)	1.8 (−2.0–5.6)	0.36
36 mo	105	41.7 (37.9–45.6)	−1.6 (−5.6–2.3)	0.42
**Fullness, mm**				
0 mo	136	33.8 (29.6–38)	–	–
2 mo	135	34.0 (29.7–38.3)	0.2 (−4–4.4)	0.93
6 mo	121	33.1 (28.7–37.5)	−0.7 (−5.7–4.2)	0.77
12 mo	119	34.1 (29.4–38.8)	0.2 (−4.7–5.1)	0.93
24 mo	114	36.3 (31.9–40.8)	2.5 (−2.4–7.4)	0.31
36 mo	105	39.3 (34.4–44.2)	5.4 (0.2–10.7)	0.04

a *P-values for the linear mixed model (0–36 months) without an interaction term for the weight-maintenance diets [i.e., P-value for the difference from pre-weight loss (0-month) values; per test α = 0.01 after Bonferroni correction]*.

### Fasting Plasma Gut Hormone Concentrations and Appetite Sensations

Intention-to-treat analyses revealed no evidence of any overall difference between the weight-maintenance diets for fasting plasma concentrations of either gut hormone over the course of the trial (*P* = 0.95 and *P* = 0.22 for ghrelin and peptide YY, respectively) (Table 2 and Figures 2B,C). During the weight-reducing phase, there was an increase from pre-weight-loss values (i.e., from 0-month values) in fasting plasma ghrelin concentrations, as well as a decrease in fasting plasma peptide YY concentrations (all *P* < 0.001) (Table 3). Moreover, the differences from 0-month values in fasting plasma peptide YY and ghrelin concentrations were attenuated within 6 and 24 months, respectively (*P* = 0.32 and *P* = 0.08, respectively, vs. 0 months) (Table 3). In terms of fasting appetite sensations, there was no evidence of any differences between the two weight-maintenance diets (all *P*-values for the interaction were >0.05, as shown in Table 2), and there were also no clear overall changes from pre-weight-loss values (i.e., from 0-month values) (Table 3).

### Weight and Fasting Gut Hormone Correlations

There was no correlation between changes in ghrelin or peptide YY during the weight-reducing phase and changes in weight during the weight-reducing phase or the subsequent weight-maintenance phase (data not shown). During the weight-maintenance phase, however, there was an inverse correlation between the change in ghrelin and the change in weight, with a Spearman correlation coefficient of −0.47 (*P* < 0.001). In other words, the greater the weight regain during the weight-maintenance diets, the greater the attenuation in ghrelin concentrations.

## Discussion

In this study, we observed that a 2-month weight-reducing diet using total meal replacement induced an increase in fasting plasma concentrations of the “hunger hormone” ghrelin and a decrease in that of the “satiety hormone” peptide YY, with no change from pre-weight-loss values in appetite sensations. However, there were no differences in gut hormone concentrations or appetite sensations between the two 34-month weight-maintenance diets differing in protein content and GI. In both weight-maintenance diets, the changes in plasma gut hormone concentrations induced by weight loss were attenuated within 6–24 months, without full weight regain.

Several trials have observed alterations in circulating concentrations of appetite-regulating gut hormones such as ghrelin and peptide YY after weight loss and during weight maintenance in adults with overweight and obesity (16–20). In these trials, ghrelin responses are rather consistent in that most trials showed statistically significant increases in fasting and post-prandial ghrelin concentrations after weight loss (17–20), while only one trial showed no statistically significant changes from pre-weight-loss values (16). In contrast, published results for peptide YY are more equivocal, as researchers have observed various responses to weight loss: a decrease in both fasting and postprandial concentrations (17), a decrease in fasting concentrations with no change in post-prandial concentrations (16), a decrease in fasting concentrations with an increase in post-prandial concentrations (18), or no changes in either fasting or post-prandial concentrations of peptide YY after weight loss (19). These hormonal changes led to the general hypothesis that “compensatory mechanisms” of weight loss-induced increases in ghrelin, with or without decreases in peptide YY, could be drivers of weight regain. Furthermore, while some of these trials showed sustained changes in appetite sensations and gut hormone concentrations that were still apparent when measured at 1 and 3 years after weight loss (17–20), the degree to which these sustained changes were attenuated during weight maintenance after weight loss varied between appetite sensations, hormones, and trials (16–20). For instance, while some trials showed no attenuation of the weight loss-induced increases in hunger (16, 17, 19, 20) or hormonal changes (17, 19) during a weight-maintenance phase, others showed partial attenuation of the increased ghrelin (18, 20) or reduced peptide YY concentrations (18, 20) during weight maintenance.

In line with some of the above-mentioned findings, we too showed that gut hormone responses to weight loss could be temporary, as we observed an attenuation of the changes, with values similar to pre-weight-loss values after 6–24 months. However, we hypothesized that even though physiological changes due to weight loss might occur, potentially causing participants to feel more hungry and thus more prone to weight regain, a higher-protein/low-GI diet might attenuate these weight loss-induced changes. Yet, we did not observe any differences in appetite sensations or gut hormone concentrations between the two weight-maintenance diets under investigation in our trial. This finding is at apparent odds with studies showing that a higher-protein/low-carbohydrate (23, 26) or lower-carbohydrate/low-GI diet (30) is effective in reducing body weight (23, 26, 30), reducing appetite sensations (23), and regulating circulating gut hormone concentrations (23, 30). The Diogenes study demonstrated that a combined higher-protein (25 vs. 13% of energy as protein) and low-GI weight-maintenance diet was the most effective for body weight maintenance during the 26-week intervention, compared to diets with either or both of a lower protein content or higher GI (26). Similarly, a 20% higher protein intake (18 vs. 15% of energy as protein) during weight maintenance caused higher sensations of satiety and a 50% lower weight regain 3 months after weight loss (23). However, in line with our findings, a recent trial, published in 2020, showed that two isoenergetic weight-maintenance diets that differed in protein, fiber, and fat content (a “higher-satiety diet” vs. a “lower-satiety diet”) were no different from each other in terms of effects on fasting gut hormone concentrations (31). A reason that may explain the discrepancy in findings might be the absolute protein content of the weight-maintenance diets (22) and differences in compliance (32). Specifically, although the target protein content of our moderate protein diet, as a percent of energy (15%), was similar to that of other studies (23, 26), the actual protein intake was ~19% of energy intake (32). Indeed, the moderate protein group in our study showed a protein intake above 0.8 g per kg of body weight, compared with 0.6 g per kg body weight in other studies (22, 23, 26). Similarly, in the recent study mentioned above (31), the percent of energy intake as protein was also around 17% in both groups, equivalent to ~0.8 g per kg of body weight, based on average pre-weight-loss body weights. According to Soenen et al. (22), a dietary protein intake of 0.8 g per kg of body weight is sufficient for body weight maintenance. This may explain the lack of differences between our two weight-maintenance diets in terms of weight change, appetite, and gut hormone concentrations.

As changes in circulating gut hormone concentrations during weight loss have been hypothesized to explain the difficulty in maintaining a diet-induced reduction in body weight, we expected to find statistically significant correlations between hormonal changes during the weight-reducing diet and the amount of weight regained during the weight-maintenance diets in this trial. Instead, we observed no correlation between hormonal changes during the weight-loss diet and subsequent weight regain, implying that changes in circulating concentrations of gut hormones may not predict the extent of weight regain after weight loss and refeeding. This was first suggested by Nymo and colleagues in 2018 (33), leading that team to hypothesize that gut hormone changes during weight loss could be viewed not as a compensatory mechanism to restore body weight, but instead as a normalization toward values seen in people of a healthy weight, as recently reviewed by Martins et al. (34). Indeed, adults with overweight and obesity have been shown to have lower circulating concentrations of ghrelin and perturbed concentrations of peptide YY compared to adults with a normal weight (19, 35, 36). Recently, DeBenedictis et al. (37) showed an increase in plasma ghrelin concentrations in adults with overweight or obesity after weight loss, to values that were not discernibly different from people of normal weight. This finding provided further support for the hypothesis that gut hormone changes after weight loss—at least for ghrelin—might be adiposity signals rather than compensatory signals. In other words, lower circulating ghrelin concentrations signal higher adiposity, with concentrations being “restored” to higher values upon weight loss, rather than an increase in ghrelin concentrations upon weight loss being compensatory signals that promote weight regain. In keeping with the hypothesis of ghrelin as an adiposity signal, we observed that weight regain during the weight-maintenance diets was correlated with a reduction of fasting plasma ghrelin concentrations during the same time, indicating that weight regain may contribute to the reestablishment of pre-weight-loss ghrelin concentrations. In other words, the changes in body weight during the weight reduction and weight-maintenance phases may have contributed to the observed changes in plasma hormone concentrations, rather than changes in hormone concentrations contributing to changes in body weight. It is therefore likely that the partial weight regain observed in our trial during the weight-maintenance diets (48.7%) was not mediated by the gut hormone changes with weight loss, but might instead be driven by other biological, behavioral, or environmental factors (37).

Our current observations of lack of any increase in fasting appetite sensations during the weight-loss diet (2 months) are in line with past research showing no change or a reduction in hunger while on a total meal replacement diet (38). However, our findings of this lack of increase in appetite sensations persisting into the weight-maintenance phase contrast with others who have shown consistent increases in both fasting and postprandial hunger sensations during a weight-maintenance phase after weight loss (16, 17, 19, 20). Only one research team has repeatedly shown an increase in postprandial fullness concomitant with increased postprandial hunger following refeeding (19, 37). Causes of conflicting findings between studies might be due to methodological factors such as the difficulty in using a visual analog scale to accurately measure appetite sensations (39). Additionally, we only focused on fasting appetite sensations in our study, which limits our view on appetite regulation. Moreover, the complexity of the appetite-regulation system and the different mechanistic pathways involved might also cause individuals to react differently to energy restriction and weight maintenance and might also be a likely explanation of the equivocal findings (40, 41).

Further to the lack of evidence for any differences between the weight-maintenance diets tested in this randomized controlled trial, our study has important clinical implications. We showed that a weight-reducing diet is not necessarily associated with an increase in appetite sensations and that changes in plasma concentrations of appetite-regulating gut hormones during weight loss (at least the two key hormones investigated in this study—ghrelin and peptide YY—other hormones, such as leptin, were not investigated) may approach pre-weight-loss levels within 6–24 months without regain of all the weight that was lost but do not appear to drive weight regain. It is likely that other biological, behavioral, or environmental factors are involved in weight regain. Thus, while people with obesity may experience biological changes in appetite regulation when losing weight, these changes may reflect a restoration of biology to that associated with a healthier body weight. In other words, these biological changes may not be the cause of weight changes and consequently might not necessarily prevent maintenance of the reduced body weight.

In conclusion, in this study of the long-term (up to 3-year) effects of weight-maintenance diets on appetite sensations and appetite-regulating hormones, we did not observe any difference between the two weight-maintenance diets under investigation (i.e., a higher-protein/low-GI diet vs. a moderate-protein/medium-GI diet).

## Data Availability Statement

The original contributions presented in the study are included in the article, further inquiries can be directed to the corresponding author.

## Ethics Statement

The studies involving human participants were reviewed and approved by The University of Sydney Human Research Ethics Committee (Protocol No X14-0408 and No 2013/535). The patients/participants provided their written informed consent to participate in this study.

## Author Contributions

AR, MF, and JB-M designed the clinical trial of the PREVIEW Study. RS and AS formulated the sub-study research question and designed the sub-study. RM and SB acquired and provided the general data for the Sydney participants, with RS, SM, JD, JZ, AD, and AW-T collecting the data for this sub-study. MB and SM performed the radioimmunoassays and collected and checked the data, with mentoring from RS and AS. MB performed the analyses and drafted the manuscript together with RS and AS. All authors critically revised the manuscript and approved of the final version to be published.

## Conflict of Interest

RS serves on the Nestlé Health Science Optifast^®^ VLCDTM™ Advisory Board. JB-M is the President of the Glycemic Index Foundation (a non-profit food endorsement program), oversees a glycemic index testing service at the University of Sydney and is the author of books about the glycemic index, food and healthy eating. AS owns 50% of the shares in Zuman International, a company which receives royalties for books she has written about weight management and payments for presentations at industry conferences. She has also received presentation fees and travel reimbursements from Eli Lilly and Co, the Pharmacy Guild of Australia, Novo Nordisk, the Dietitians Association of Australia, Shoalhaven Family Medical Centres, the Pharmaceutical Society of Australia, and Metagenics, and served on the Nestlé Health Science Optifast VLCD advisory board from 2016 to 2018. FA is a director of the Glycemic Index Foundation (a non-profit food endorsement program), manages a glycemic index testing service at the University of Sydney, and is a co-author of books about the glycemic index. Finally, Cambridge Weight Plan^©^, Ltd, UK provided all meal replacement products used at all sites of the PREVIEW Study. The commercial and funding sponsors had no role in the design of the study; in the collection, analyses, or interpretation of data; in the writing of the manuscript, nor in the decision to publish the results. The remaining authors declare that the research was conducted in the absence of any commercial or financial relationships that could be construed as a potential conflict of interest.

## Abbreviations

CHO: carbohydrate
GI: glycemic index
HP/LGI: higher protein/low glycemic index
MP/MGI: moderate protein/moderate glycemic index.
